# From the Sound of Silence to Ultrasound of Life?^☆^

**DOI:** 10.1016/j.jped.2025.101483

**Published:** 2025-12-04

**Authors:** Samuel Kunz, Luregn Jan Schlapbach, Fábio Joly Campos

**Affiliations:** [1] Department of Intensive Care and Neonatology, and Children’s Research Center, University Children's Hospital Zurich, University of Zurich, Zurich, Switzerland; [2] Child Health Research Centre, The University of Queensland, Brisbane, Australia; [3] Botucatu Faculty of Medicine, Department of Pediatrics, Universidade Estadual Paulista Júlio de Mesquita Filho (UNESP), Botucatu, Sao Paulo, SP, Brazil

Volume-overload in critically ill patients is associated with higher mortality and morbidity [1,2]. Therefore, a thorough knowledge of each patients’ fluid-state is essential to direct organ support but remains challenging. Traditional bedside assessments such as presence of oedema, liver size, crackles on lung auscultation, oxygenation, and weight gain remain cornerstones of clinical examination but lack sensitivity and specificity, in particular in critically ill, often comorbid pediatric ICU patients [3]. Invasive assessments of lung fluid state, such as extravascular lung-water (EVLW) index can be measured through thermodilution and have been shown to be associated with severity, and lower EVLW indices were observed in survivors [4]. However, most adult studies have not demonstrated a benefit of more invasive monitoring [5], and the methods have been even less widely used, nor rigorously validated in critically ill children [6].

Less invasively, EVLW can be estimated by lung ultrasound [7]. Lung ultrasound has several advantages over X-rays and is associated with less efforts, resources, and exposure to radiation compared to computer tomography scans. Another advantage of lung ultrasound is that changes in pulmonary fluid status can be detected rapidly at the bedside, with an increase in the EVLW score observed already after as little as one fluid bolus [8]. Indeed, small single center observational studies indicate potential association of EVLW scores with clinical assessments of respiratory effort. This is highly relevant as optimal cardiovascular support and titration of fluid treatment, as well as active fluid removal, remain one of the most controversial topics in pediatric critical care medicine. The challenge applies to various clinical scenarios, such as sepsis and septic shock, Acute Respiratory Distress Syndrome, burns, or recovery post cardiopulmonary bypass. The challenges are magnified in oncology patients, where fluid overload, pulmonary oedema, and increased risk of infection remain common complications of anti-cancer treatment toxicity [9].

In 2017, El-Nawawy et al. performed one of the first randomized trials in the field [10] and demonstrated that by monitoring pediatric patients with septic shock with bedside ultrasound, there was a decrease in the time to shock reversal. One of the possible explanations of the effect relates to better, more personalized titration of fluids administered, possibly reducing fluid overload. Two important concepts to consider include fluid responsiveness and fluid tolerance. Fluid responsiveness is the ability to improve cardiac output by administering fluid, and fluid tolerance is the individual’s capacity to receive fluid without leading to extravasation into the extravascular space. However, this approach often requires considerable training for the examiner, and multiple assessments of flow velocities of the hepatic, portal, and renal veins, in addition to the diameter of the vena cava. One of the most common approaches study fluid tolerance with ultrasound has been the VEXUS score [4].

In this context, the recent paper by Camargo et al. [11] provides valuable addition to the current knowledge. How can we better understand changes in lung ultrasound after fluid administration and correlate them with clinical outcomes, such as ICU length of stay and mortality? The authors included 83 pediatric oncology patients admitted to a single center Brazilian PICU in a prospective cohort study conducted over 21 months. Exclusion criteria were congenital heart disease, significant intracardiac shunts, an ejection fraction below 50% or severe arrhythmias and patients with lung ultrasound already indicating a hypervolemic state before volume-expansion. A total of 88 datasets were generated on patients receiving 10 ml/kg saline 0.9% fluid bolus over 30 minutes. Lung ultrasound was performed by an experienced operator immediately before and after the fluid bolus. Additionally, echocardiography, and central venous blood gas analysis was performed. The patients included a range of oncological conditions, such as hematopoietic stem cell transplants (23%), acute leukemias (16%), or various brain tumors.

The authors observed a significant increase of B-lines after the fluid administration indicating rapid detectability of EVLW changes with lung ultrasound. The mean number of B-lines was an independent predictor of ICU length of stay and death in multivariate prognostic models. Of interest, the strength of the association in terms of adjusted effect sizes was comparable to elevated lactate levels, a well-established risk factor of death [12,13]. The study’s endpoints were well chosen, objective and clinically relevant. The authors pragmatically chose to reduce the intercostal spaces examined on LUS from 28 to 12, supported by previous findings, albeit this could have reduced sensitivity for subtle EVLW changes [14,15]. The authors are to be commended for performing a well-designed observational study on an understudied high-risk population allowing to evaluate the diagnostic value of lung ultrasound before and after a standardized fluid challenge with clinical and physiological parameters. The results implicate that lung ultrasound is a promising and easy-to-use bedside diagnostic tool to estimate fluid balance and fluid overload in critically ill children. Nevertheless, further evaluation in bigger patient collectives and subgroups is needed, and additional comparison to other ultrasound-guided estimates like Venous Excess Ultrasound (VExUS) are desirable.

A number of limitations are worth highlighting, as these may serve to direct the design of future studies in the field. First, the indication for the fluid bolus remains unclear – at baseline only a minority of patients were on hemodynamic support or considered to be in shock. Interestingly, a response to fluid in terms of increase in the cardiac index was observed in only about half of patients, consistent with previous reports showing very variable and usually not sustained impact of fluid bolus on hemodynamic status [16,17]. Second, concomitant therapies such as vasoactive support, ongoing active fluid removal, and extent of invasive respiratory support could have affected study findings. Third, the single center design, moderate sample size, and lack of comparison groups such as postoperative cardiac patients limits which conclusions can be drawn about subgroups, or which patients may benefit the most from lung ultrasound. Fourth, both lung ultrasound and echocardiography are examiner-dependent examinations, and inter-operator variability could have been assessed by repeat observations by different examiners to rule out measurement bias. Finally, as lung ultrasound is used for estimates of extravascular water-overload, VeXUS is well established as a tool to assess intravascular fluid state. The advantages and disadvantages of either of the tools, whether their combination provide actionable added value for patient management, optimal patient selection, and therapeutic implications need to be elaborated in future studies.

Ideally, a novel diagnostic device or marker would meet requirements for validity, efficacy, and generalizability. While the *construct validity* (does lung ultrasound measure pulmonary fluid state?) is based on physical properties of the technology supporting its *face validity* (is lung ultrasound suitable to assess lung fluid status rapidly at the bedside?), *content validity* (does lung ultrasound provide representative data on overall fluid status?) is less well studied, and *criterion validity* (do lung ultrasound findings, such as EVLW scores, accurately measure lung fluid status?) remains based on consensus statements for pediatric age groups. For example, the cut-off for a pathological number of B-lines, along with the localization and number of acoustic sites, has not yet been clearly established, especially in children. Moreover, larger studies investigating the robustness of the association of EVLW estimates by lung ultrasound with clinical outcomes are needed. The recent ESICM/ESPNIC expert consensus-based guidelines [14] on the use of lung ultrasound provide valuable direction and serve to support local implementation, teaching, best practice, and hopefully enhance the standardization of future studies. In terms of *efficacy*, the history of ICU research and practice repeatedly struggled to demonstrate patient-centered benefits of novel diagnostic modalities, despite their promises to clinicians and based on positive preliminary findings from non-randomized study designs. Given the rapid uptake of point-of-care ultrasound by the adult, pediatric, and neonatal ICU community, and its potential to improve diagnostic yield, shorten time to clinically actionable information, and reduce conventional diagnostics such as X-rays, future trials could investigate whether structured application of POCUS interventions such as lung ultrasound provide benefits to management. Finally, *generalizability* remains little investigated, with most lung ultrasound studies being single center, questioning the dependence of findings on operator training and experience, patient selection, clinical context within the unit setting, epidemiology, and POCUS device. Given the renewed interest to study fluid-sparing approaches in critically ill children, in particular in the field of sepsis [18], trials investigating the role of lung ultrasound and other POCUS tools to enable pragmatic yet personalized patient management are thus urgently needed [19]. Importantly, commercialization of bedside ultrasound devices and technological advances in size, cost, and portability, carry promise to make this technology more widely available, including low and middle income country settings.

In summary, lung ultrasound seems to be a promising non-invasive tool for fluid-state assessment of critically ill children, but indications, diagnostic and treatment algorithms, generalizability, and clinical implications still require further study.

## Funding

LJS received support from the NOMIS and the Thomas and Doris Ammann Foundation.

## Data availability

Not applicable.

## Declaration of competing interest

The authors have nothing to declare.
